# COVID-19 – The Availability of ICU Beds in Brazil during the Onset of Pandemic

**DOI:** 10.5334/aogh.3025

**Published:** 2020-08-13

**Authors:** Camila Vantini Capasso Palamim, Fernando Augusto Lima Marson

**Affiliations:** 1 Laboratory of Cell and Molecular Tumor Biology and Bioactive Compounds, São Francisco University, Bragança Paulista, São Paulo, BR; 2 Laboratory of Human and Medical Genetics, São Francisco University, Bragança Paulista, São Paulo, BR

## Abstract

**Background:**

Brazil faces some challenges in the battle against the COVID-19 pandemic, including: the risks for cross-infection (community infection) increase in densely populated areas; low access to health services in areas where the number of beds in intensive care units (ICUs) is scarce and poorly distributed, mainly in states with low population density.

**Objective:**

To describe and intercorrelate epidemiology and geographic data from Brazil about the number of intensive care unit (ICU) beds at the onset of COVID-19 pandemic.

**Methods:**

The epidemiology and geographic data were correlated with the distribution of ICU beds (public and private health systems) and the number of beneficiaries of private health insurance using Pearson’s Correlation Coefficient. The same data were correlated using partial correlation controlled by gross domestic product (GDP) and number of beneficiaries of private health insurance.

**Findings:**

Brazil has a large geographical area and diverse demographic and economic aspects. This diversity is also present in the states and the Federal District regarding the number of COVID-19 cases, deaths and case fatality rate. The effective management of severe COVID-19 patients requires ICU services, and the scenario was also dissimilar as for ICU beds and ICU beds/10,000 inhabitants for the public (SUS) and private health systems mainly at the onset of COVID-19 pandemic. The distribution of ICUs was uneven between public and private services, and most patients rely on SUS, which had the lowest number of ICU beds. In only a few states, the number of ICU beds at SUS was above 1 to 3 by 10,000 inhabitants, which is the number recommended by the World Health Organization (WHO).

**Conclusions:**

Brazil needed to improve the number of ICU beds units to deal with COVID-19 pandemic, mainly for the SUS showing a late involvement of government and health authorities to deal with the COVID-19 pandemic.

## Background

In the third week of April 2020, the number of COVID-19 cases reached the two million mark around the world. In nearly four months, the disease caused the death of ~153,920 people. During the onset of the COVID-19 pandemic in Brazil, a total of 569,953 individuals (14,0246 patients in Brazil) have recovered from COVID-19: this number represents four times the number of global deaths. So far, 56,924 individuals (6,634 patients in Brazil) have received intensive therapy due to the high severity of the disease. From the first identified case, Brazil has reached a total of 33,682 confirmed cases of the disease and 2,411 deaths, with both marks ranking 11th globally. In total, 210 countries and territories as well as two international conveyances worldwide were affected by the disease; and deaths caused by COVID-19 occurred in 166 countries and territories at this moment [1,2].

To date, the 17th July 2020, the number of COVID-19 cases reached the 14 million mark around the world causing the death of ~595,166 people. At Brazil, a total of 2,021,834 individuals were affected by COVID-19 and the disease caused the death of 76,997 individuals with both marks ranking 2nd globally. Brazil had 578,062 active cases ranking 2nd globally, 9,509 total cases of COVID-19 per one million of inhabitants ranking 15th globally and 362 deaths caused by COVID-19 per one million of inhabitants ranking 12th globally [1,2]. In addition, Brazil performed only 4,911,06 SARS-CoV-2 real time polymerase chain reaction (RT-PCR) tests ranking 9th globally and the ratio between the number of SARS-CoV-2 RT-PCR tests done per one million of inhabitants was 23,097 ranking 104th globally [1,2]. To date, in total, 213 countries and territories as well as two international conveyances worldwide were affected by the COVID-19 pandemic; and deaths caused by the disease occurred in 187 countries and territories at this moment [1,2]. A summary of the COVID-19 pandemic scenario from Brazil by state and Federal District on 17 July 2020 was shown as Table 1.

**Table 1:** Demographic characteristics of COVID-19 at the Brazil in 17^th^ July 2020.

States and the Federal District	Cases	Deaths	Case Fatality Rate	Cases/1 M	Deaths/1M

**Brasil**	2,012,151	76,688	3.8	957.5	36.5
**Midwest region**	164,263	3,421	2.1	1,007.9	21
Goiás	40,201	986	2.5	572.8	14
Mato Grosso	31,111	1,207	3.9	892.8	34.6
Distrito Federal	77,621	1,037	1.3	2,574.3	34.4
Mato Grosso do Sul	15,330	191	1.2	551.6	6.9
**South region**	144,530	2,975	2.1	482.2	9.9
Santa Catarina	49,781	588	1.2	694.8	8.2
Rio Grande do Sul	45,344	1,141	2.5	398.6	10
Paraná	49,405	1,246	2.5	432.1	10.9
**North region**	340,656	10,790	3.2	1,848.3	58.5
Acre	16,865	447	2.7	1,912.3	50.7
Rondônia	28,654	677	2.4	1,612.3	38.1
Tocantins	16,672	278	1.7	1,060	17.7
Amazonas	88,025	3,095	3.5	2,123.8	74.7
Amapá	33,004	493	1.5	3,902.4	58.3
Pará	133,039	5,385	4.0	1,546.4	62.6
Roraima	24,397	415	1.7	4,027.5	68.5
**Northeast region**	673,493	24,645	3.7	1,180.1	43.2
Alagoas	48,734	1,348	2.8	1,460.3	40.4
Pernambuco	76,091	5,836	7.7	796.2	61.1
Bahia	116,373	2,693	2.3	782.4	18.1
Paraíba	65,423	1,418	2.2	1,628.2	35.3
Sergipe	41,226	1,071	2.6	1,793.5	46.6
Piauí	36,542	1,043	2.9	1,116.4	31.9
Ceará	144,000	7,127	4.9	1,576.9	78
Maranhão	104,126	2,608	2.5	1,471.7	36.9
Rio Grande do Norte	40,978	1,501	3.7	1,168.5	42.8
**Southest region**	689,209	34,857	5.1	779.9	39.4
São Paulo	402,048	19,038	4.7	875.6	41.5
Espírito Santo	68,118	2,136	3.1	1695	53.2
Rio de Janeiro	134,573	11,849	8.8	779.5	68.6
Minas Gerais	84,470	1,834	2.2	399	8.7

The data was collected at https://covid.saude.gov.br. Access July 17, 2020. Reference 3.

Brazil has a vast geography and includes 26 states and one Federal District. The states and the Federal District have different numbers of inhabitants and an uneven geographical distribution of the population, causing a heterogeneous population density (persons per km^2^) (Table 2). In each state or the Federal District, epidemiologic data for COVID-19 showed different numbers of patients and/or deaths and case fatality rate (CFR). During the onset of COVID-19 pandemic most cases were reported in the state of São Paulo, namely 12,841/33,682 patients, 928/2,411 deaths and CFR of 7.2%. At this moment, the CFR for COVID-19 in Brazil ranged from 1.8% (state of Roraima) to 13.3% (state of Paraíba) [3]. Brazil had seven states with CFR higher than the global mean CFR. In addition, the Brazilian gross domestic product (GDP) differs among the states and the Federal District due to different levels of economic development, and consequently affecting the capacity and quality of health services in each region. Brazil showed lower numbers of COVID-19 cases in 15 states and the Federal District and higher numbers in 10 other states. In one state, the number of COVID-19 cases was identical to that of countries with similar number of inhabitants. Thus, Brazil faces some challenges in the battle against the COVID-19 pandemic, including: the risks for cross-infection (community infection) increase in densely populated areas; low access to health services in areas where the number of beds in intensive care units (ICUs) is scarce and poorly distributed, mainly in states with low population density.

**Table 2:** Demographic characteristics of COVID-19 in the Brazil including gross domestic product (GDP), number of inhabitants, area of each state or the Federal District, population density and countries with similar number of inhabitants.

States and the Federal District	Number of patients with COVID-19	Deaths due to COVID-19	CFR	GDP (R$) × 10^3^	Number of inhabitants	% of the population	Area (km^2^)	Population density (persons per km^2^)	Countries with similar number of inhabitants (Number of COVID-19 cases and deaths)

Acre	135	5	3.7	R$ 13,751	881,935	0.4	164,123.74	5.37	Fiji (859,178) – 17/0
Alagoas	110	7	6.4	R$ 49,456	3,337,357	1.6	27,843.30	119.86	Uruguay (3,415,866) – 502/9
Amapá	370	10	2.7	R$ 14,339	845,731	0.4	142,470.76	5.94	Fiji (859,178) – 17/0
Amazonas	1,809	145	8	R$ 89,017	4,144,597	2	1,559,168.12	2.66	Lebanon (4,168,000) – 668/21
Bahia	1,059	36	3.4	R$ 258,649	14,873,064	7.1	564,722.61	26.34	Chad (14,037,000) – 27/0
Ceará	2,684	149	5.6	R$ 138,379	9,132,078	4.3	148,894.76	61.33	United Arab Emirates (9,157,000) – 6,300/37
Distrito Federal	746	20	2.7	R$ 235,497	3,015,268	1.4	5,760.78	523.41	Lithuania (2,900,787) – 1,150/33
Espírito Santo	856	25	2.9	R$ 109,227	4,018,650	1.9	46,074.44	87.22	Lebanon (4,168,000) – 668/21
Goiás	335	16	4.8	R$ 181,692	7,018,354	3.3	340,125.72	20.63	Paraguay (7,003,406) – 199/8
Maranhão	797	40	5	R$ 85,286	7,075,181	3.4	329,642.17	21.46	Paraguay (7,003,406) – 199/8
Mato Grosso	162	5	3.1	R$ 123,834	3,484,466	1.7	903,207.00	3.86	Uruguay (3,415,866) – 502/9
Mato Grosso do Sul	143	5	3.5	R$ 91,866	2,778,986	1.3	357,145.54	7.78	Jamaica (2,717,991) – 143/5
Minas Gerais	1,021	35	3.4	R$ 544,634	21,168,791	10.1	586,521.12	36.09	Sri Lanka (20,675,000) – 244/7
Paraná	874	42	4.8	R$ 401,662	11,433,957	4.1	199,305.24	57.37	Austria (8,602,112) – 14,600/431
Paraíba	195	26	13.3	R$ 59,089	4,018,127	1.9	56,467.24	71.16	Lebanon (4,168,000) – 668/21
Pará	557	26	4.7	R$ 138,068	8,602,865	5.4	1,245,759.31	6.91	Bolivia (11,410,651) – 465/31
Pernambuco	2,006	186	9.3	R$ 167,290	9,557,071	4.5	98,068.02	97.45	Byelorussia (9,485,300) – 4,780/42
Piauí	102	8	7.8	R$ 41,406	3,273,227	1.6	251,616.82	13.01	Haiti (3,268,431) – 43/3
Rio Grande do Norte	463	23	5	R$ 59,661	3,506,853	1.7	52,809.60	66.41	Uruguay (3,415,866) – 502/9
Rio Grande do Sul	802	22	2.7	R$ 408,645	11,377,239	5.4	281,707.15	40.39	Belgium (11,250,659) – 36,140/5,163
Rio de Janeiro	4,349	341	7.8	R$ 640,186	17,264,943	8.2	43,750.42	394.62	Netherlands (16,922,900) – 30,450/3,459
Rondônia	92	3	3.3	R$ 39,451	1,777,225	0.8	237,765.23	7.47	Gabon (1,725,000) – 108/1
Roraima	164	3	1.8	R$ 11,011	605,761	0.3	224,273.83	2.7	Luxemburg (562,958) – 3,480/72
Santa Catarina	926	30	3.2	R$ 256,661	7,164,788	3.4	95,730.92	74.84	Serbia (7,114,393) – 5,690/110
Sergipe	53	4	7.5	R$ 38,867	2,298,696	1.1	21,926.91	104.83	Namibia (2,280,700) – 16/0
São Paulo	12,841	928	7.2	R$ 2,038,005	45,919,049	21.9	248,219.48	184.99	Spain (46,439,864) – 190,840/20,002
Tocantins	31	1	3.2	R$ 31,576	1,572,866	0.7	277,720.40	5.66	Bahrain (1,359,800) – 1,740/7

*Note*: CFR – Case Fatality Rate. The GDP for 2017 was used. The number of number of inhabitants, % of the population in each state or the Federal District and population density were estimated in 2019. The data was collected at https://www.who.int/emergencies/diseases/novel-coronavirus-2019. [Reference 1]; https://www.worldometers.info/coronavirus/. [Reference]. https://covid.saude.gov.br. [Reference 3]. Access July 17, 2020.

In Brazil, the majority of the population receives primary care from the Brazilian Public Health System (SUS), and yet the private health system maintains the major number of ICU beds. In some cases, the state can request support from the private healthcare sector. There is a difference in the number of available ICU beds among the states and the Federal District, as well as between the public and the private health systems. According to the World Health Organization (WHO), the number of ICU beds should be 1 to 3 units per 10,000 inhabitants. In Brazil, at least one ICU bed per 10,000 inhabitants was reported in 24 states and in the Federal District (except for the states of Acre and Roraima). However, these numbers were calculated by adding ICU beds from the public and the private health services.

Considering the number of ICU beds at SUS/10,000 inhabitants, only 11 states showed a number of ICUs beds above one ICU bed per 10,000 inhabitants. Moreover, considering only the beneficiaries of private health insurance and the number of ICU beds at the private health system/10,000 inhabitants, all states show a number of ICU beds above three ICU beds per 10,000 inhabitants, except for the state of Santa Catarina, where a total of 2.61 ICUs beds per 10,000 inhabitants were described. The highest number of ICU beds per 10,000 inhabitants was found in the state of Mato Grosso, i.e., a rate of 10.63. São Paulo, the most populous state in Brazil, has 1.19 and 3.8 ICUs beds/10,000 inhabitants at the public (SUS) and private health systems, respectively. Moreover, each state shows an uneven distribution of ICUs beds per total area, for example, Amazonas is the largest state in Brazil with 559,168.12 km^2^ and ICUs were concentrated only in its capital city, Manaus (Table 3).

**Table 3:** Distribution of intensive care unit (ICU) beds per state or the Federal District regarding public and private health systems.

States and the Federal District	ICU beds	Ratio of ICU beds to total beds (%)	ICU beds/10,000 inhabitants	ICU beds at the Public Health System (SUS)	Ratio of ICU beds to total beds (%) at SUS	ICU beds at SUS/10,000 inhabitants	Beneficiaries of private health insurance	ICU beds at the Private Health System	Ratio of ICU beds to total beds (%) at the Private Health System	ICU beds at the Private Health System/10,000 inhabitants

Acre	75	0.2	0.9	59	0.3	0.71	45,245	16	0.07	3.54
Alagoas	491	1.1	1.45	292	1.4	0.86	384,134	199	0.87	5.18
Amapá	82	0.2	1.03	26	0.1	0.33	67,717	56	0.25	8.27
Amazonas	502	1.1	1.24	321	1.5	0.79	527,803	181	0.8	3.43
Bahia	2,029	4.6	1.32	988	4.6	0.64	1,603,515	1,041	4.58	6.49
Ceará	1,201	2.7	1.33	690	3.2	0.76	1,274,740	511	2.25	4.01
Distrito Federal	1,031	2.3	3.39	270	1.3	0.89	866,861	761	3.35	8.78
Espírito Santo	1,091	2.5	2.72	478	2.2	1.19	1,094,094	613	2.69	5.6
Goiás	1,409	3.2	2.08	751	3.5	1.11	1,112,306	658	2.89	5.92
Maranhão	787	1.8	1.12	410	1.9	0.59	461,132	377	1.66	8.18
Mato Grosso	877	2	2.62	297	1.4	0.89	545,501	580	2.55	10.63
Mato Grosso do Sul	484	1.1	1.78	254	1.2	0.94	488,152	230	1.01	4.71
Minas Gerais	4,341	9.8	2.06	2,742	12.7	1.3	5,093,159	1,599	7.03	3.14
Paraná	2,858	6.5	2.52	1,748	8.1	1.54	2,822,209	1,110	4.88	3.93
Paraíba	608	1.4	1.51	378	1.8	0.94	419,264	230	1.01	5.49
Pará	984	2.2	1.18	474	2.2	0.57	794,273	510	2.24	6.42
Pernambuco	1,861	4.2	1.96	1,034	4.8	1.09	1,311,470	827	3.64	6.31
Piauí	353	0.8	1.1	179	0.8	0.56	312,534	174	0.76	5.57
Rio Grande do Norte	601	1.4	1.71	330	1.5	0.94	520,844	271	1.19	5.2
Rio Grande do Sul	2,374	5.4	2.1	1,506	7	1.33	2,624,252	868	3.82	3.31
Rio de Janeiro	6341	14.3	3.79	1,626	7.6	0.97	5,419,339	4,715	20.73	8.7
Rondônia	294	0.7	1.63	183	0.9	1.01	156,749	111	0.49	7.08
Roraima	48	0.1	0.92	30	0.1	0.57	28,619	18	0.08	6.29
Santa Catarina	1,108	2.5	1.58	718	3.3	1.03	1,494,526	390	1.71	2.61
Sergipe	339	0.8	1.48	230	1.1	1.01	313,827	109	0.48	3.47
São Paulo	11,863	26.8	2.63	5,358	24.9	1.19	17,126,024	6,505	28.6	3.8
Tocantins	221	0.5	1.43	134	0.6	0.86	103,966	87	0.38	8.37

*Note*: The data is based on the Federal Council of Medicine, 2018.

Given the low ICU beds availability, the healthcare system in the states of Amazonas, Pernambuco and Ceará, for example, was on the verge of collapse, as 100% of ICUs beds are being used to treat patients with COVID-19. In the state of Amazonas, the situation was critical, and the local health system was overwhelmed: high death tolls, refrigerated containers were being used to temporarily accommodate the victims’ bodies, mass graves were being dug, and burials were being conducted by family members without any infection control measures. Likewise, the public health system in the state of Rio de Janeiro was projected to collapse. The state of São Paulo, the epicenter of the crisis, was also nearing 100% capacity of ICU beds in public hospitals. However, the accurate number or projections for COVID-19 victims cannot be determined because access to SARS-CoV-2 RT-PCR was limited [4]. In this context, the epidemiology and geographic data were described and intercorrelated to evidence the need to optimize the number of ICU beds to deal with the COVID-19 pandemic during its onset.

## Data collection

The following epidemiology and geographic data markers were evaluated in our study: number of patients with COVID-19; deaths due to COVID-19; CFR for COVID-19; GDP (in Brazilian Real – R$); number of inhabitants; % of the population per area; total area of the states and Federal District (km^2^); population density (persons per km^2^); countries with similar number of inhabitants using as parameter each Brazilian state or Federal District (Table 2).

The epidemiology and geographic data were correlated with the distribution of ICU beds and the number of beneficiaries of private health insurance (Table 3) using Pearson’s Correlation Coefficient. The same data were correlated using partial correlation controlled by GDP and number of beneficiaries of private health insurance (Tables 4 and 5). The statistical analysis was done using the Statistical Package for the Social Sciences (IBM SPSS Statistics for Macintosh, Version 25.0). An alpha error of 0.05 was considered in all statistical analysis.

**Table 4:** Pearson correlation between demographic characteristics of COVID-19 and Brazil, with the distribution of intensive care unit (ICU) beds per state or the Federal District regarding public and private health systems.

Markers		Number of patients with COVID-19	Deaths due to COVID-19	CFR	GDP (R$) × 10^3^	Area (km^2^)	Population density (persons per km^2^)	Number of inhabitants	% of the population

**Number of patients with COVID-19**	Pearson correlation		0.994	0.252	0.941	–0.025	0.341	0.895	0.894
	p-value		≤0.001	0.205	≤0.001	0.901	0.082	≤0.001	≤0.001
**Deaths due to COVID-19**	Pearson correlation	0.994		0.320	0.920	–0.021	0.340	0.870	0.869
	p-value	≤0.001		0.104	≤0.001	0.919	0.082	≤0.001	≤0.001
**CFR**	Pearson correlation	0.252	0.320		0.130	–0.044	0.133	0.170	0.170
	p-value	0.205	0.104		0.519	0.829	0.508	0.396	0.396
**GDP (R$) x 10^3^**	Pearson correlation	0.941	0.920	0.130		–0.032	0.341	0.959	0.951
	p-value	≤0.001	≤0.001	0.519		0.874	0.082	≤0.001	≤0.001
**Area (km^2^)**	Pearson correlation	–0.025	–0.021	–0.044	–0.032		–0.371	0.045	0.079
	p-value	0.901	0.919	0.829	0.874		0.057	0.823	0.694
**Population density (persons per km^2^)**	Pearson correlation	0.341	0.340	0.133	0.341	–0.371		0.249	0.243
	p-value	0.082	0.082	0.508	0.082	0.057		0.210	0.223
**Number of inhabitants**	Pearson correlation	0.895	0.870	0.170	0.959	0.045	0.249		0.997
	p-value	≤0.001	≤0.001	0.396	≤0.001	0.823	0.210		≤0.001
**% of the population**	Pearson correlation	0.894	0.869	0.170	0.951	0.079	0.243	0.997	
	p-value	≤0.001	≤0.001	0.396	≤0.001	0.694	0.223	≤0.001	
**ICU beds**	Pearson correlation	0.920	0.908	0.187	0.974	–0.050	0.390	0.965	0.956
	p-value	≤0.001	≤0.001	0.351	≤0.001	0.803	0.044	≤0.001	≤0.001
**Ratio of ICU beds to total beds (%)**	Pearson correlation	0.920	0.908	0.187	0.974	–0.052	0.388	0.965	0.956
	p-value	≤0.001	≤0.001	0.352	≤0.001	0.797	0.045	≤0.001	≤0.001
**ICU beds/10,000 inhabitants**	Pearson correlation	0.379	0.373	0.002	0.472	–0.203	0.747	0.396	0.373
	p-value	0.051	0.055	0.992	0.013	0.310	≤0.001	0.041	0.055
**ICU beds at the Public Health System (SUS)**	Pearson correlation	0.865	0.840	0.141	0.962	–0.010	0.233	0.978	0.965
	p-value	≤0.001	≤0.001	0.484	≤0.001	0.962	0.243	≤0.001	≤0.001
**Ratio of ICU beds to total beds (%) at SUS**	Pearson correlation	0.866	0.841	0.142	0.962	–0.010	0.235	0.978	0.965
	p-value	≤0.001	≤0.001	0.478	≤0.001	0.961	0.237	≤0.001	≤0.001
**ICU beds at SUS/10,000 inhabitants**	Pearson correlation	0.236	0.222	0.063	0.413	–0.196	0.184	0.398	0.355
	p-value	0.237	0.265	0.755	0.032	0.328	0.358	0.040	0.069
**Beneficiaries of private health insurance**	Pearson correlation	0.947	0.929	0.157	0.995	–0.042	0.312	0.962	0.955
	p-value	≤0.001	≤0.001	0.433	≤0.001	0.836	0.113	≤0.001	≤0.001
**ICU beds at the Private Health System**	Pearson correlation	0.911	0.909	0.212	0.930	–0.079	0.488	0.901	0.896
	p-value	≤0.001	≤0.001	0.289	≤0.001	0.696	0.010	≤0.001	≤0.001
**Ratio of ICU beds to total beds (%) at the Private Health System**	Pearson correlation	0.911	0.909	0.212	0.930	–0.079	0.488	0.901	0.896
	p-value	≤0.001	≤0.001	0.289	≤0.001	0.696	0.010	≤0.001	≤0.001
**ICU beds at the Private Health System/10,000 inhabitants**	Pearson correlation	–0.159	–0.132	–0.157	–0.208	0.018	0.244	–0.247	–0.233
	p-value	0.429	0.511	0.435	0.297	0.930	0.220	0.214	0.241

*Note*: CFR - Case fatality rate; GDP - gross domestic product.

**Table 5:** Correlation controlled by gross domestic product and beneficiaries of private health insurance between demographic characteristics of COVID-19 in Brazil and the distribution of intensive care units (ICU) beds per state or the Federal District regarding the public and private health systems.

Markers		Number of patients with COVI-19	Deaths due to COVID-19	CFR	Area (km^2^)	Population density (persons per km^2^)	Number of inhabitants	% of the population

**Number of patients with COVID-19**	Partial correlation		0.960	0.325	0.048	0.172	–0.173	–0.109
	p-value		≤0.001	0.113	0.818	0.411	0.408	0.603
**Deaths due to COVID-19**	Partial correlation	0.960		0.465	0.061	0.192	–0.230	–0.168
	p-value	≤0.001		0.019	0.774	0.357	0.268	0.424
**CFR**	Partial correlation	0.325	0.465		–0.011	0.197	0.086	0.074
	p-value	0.113	0.019		0.957	0.344	0.682	0.725
**Area (km^2^)**	Partial correlation	0.048	0.061	–0.011		–0.435	0.311	0.405
	p-value	0.818	0.774	0.957		0.030	0.130	0.045
**Population density (persons per km^2^)**	Partial correlation	0.172	0.192	0.197	–0.435		–0.225	–0.213
	p-value	0.411	0.357	0.344	0.030		0.280	0.307
**Number of inhabitants**	Partial correlation	–0.173	–0.230	0.086	0.311	–0.225		0.961
	p-value	0.408	0.268	0.682	0.130	0.280		≤0.001
**% of the population**	Partial correlation	–0.109	–0.168	0.074	0.405	–0.213	0.961	
	p-value	0.603	0.424	0.725	0.045	0.307	≤0.001	
**ICU beds**	Partial correlation	–0.094	–0.013	0.176	–0.049	0.449	0.414	0.345
	p-value	0.656	0.949	0.401	0.815	0.024	0.040	0.091
**Ratio of ICU beds to total beds (%)**	Partial correlation	–0.101	–0.020	0.174	–0.057	0.443	0.414	0.342
	p-value	0.631	0.923	0.406	0.788	0.027	0.040	0.094
**ICU beds/10,000 inhabitants**	Partial correlation	–0.142	–0.091	0.013	–0.252	0.681	–0.157	–0.212
	p-value	0.499	0.667	0.952	0.224	≤0.001	0.454	0.309
**ICU beds in public health system (SUS)**	Partial correlation	–0.602	–0.609	–0.046	0.120	–0.298	0.688	0.545
	p-value	0.001	0.001	0.825	0.568	0.148	≤0.001	0.005
**Ratio of ICU beds to total beds (%) at SUS**	Partial correlation	–0.602	–0.607	–0.038	0.119	–0.291	0.689	0.545
	p-value	0.001	0.001	0.857	0.571	0.158	≤0.001	0.005
**ICU beds at SUS/10,000 inhabitants**	Partial correlation	–0.489	–0.437	0.040	–0.212	0.023	0.040	–0.109
	p-value	0.013	0.029	0.849	0.308	0.914	0.848	0.604
**ICU beds at the Private Health System**	Partial correlation	0.239	0.322	0.198	–0.114	0.604	0.029	0.039
	p-value	0.250	0.117	0.343	0.586	0.001	0.892	0.852
**Ratio of ICU beds to total beds (%) at the Private Health System**	Partial correlation	0.239	0.322	0.198	–0.114	0.604	0.029	0.040
	p-value	0.249	0.116	0.343	0.587	0.001	0.891	0.851
**ICU beds at the Private Health System/10,000 inhabitants**	Partial correlation	0.182	0.237	–0.085	–0.007	0.307	–0.124	–0.066
	p-value	0.383	0.254	0.685	0.972	0.136	0.556	0.754

*Note*: CFR - Case fatality rate.

## Main findings

Our data show a positive correlation between (i) number of patients with COVID-19 and deaths caused by the disease (CC = 0.994; *P*-value ≤ 0.001); GDP (CC = 0.941; *P*-value ≤ 0.001); number of inhabitants (CC = 0.895; *P*-value ≤ 0.001) and % of the population (CC = 0.894; *P*-value ≤ 0.001); (ii) deaths due to COVID-19 with GDP (CC = 0.920; *P*-value ≤ 0.001); number of inhabitants (CC = 0.870; *P*-value ≤ 0.001) and % of the population (CC = 0.869; *P*-value ≤ 0.001).

Moreover, (i) the number of ICUs beds and the ratio of ICU beds to total beds were correlated with the number of patients with COVID-19 (CC = 0.920; *P*-value ≤ 0.001); deaths due to COVID-19 (CC = 0.908; *P*-value ≤ 0.001); GDP (CC = 0.974; *P*-value ≤ 0.001); demographic density (CC = 0.390; *P*-value = 0.044); number of inhabitants (CC = 0.965; *P*-value ≤ 0.001) and % of the population (CC = 0.956; *P*-value ≤ 0.001); (ii) ICU beds/10,000 inhabitants was correlated with GDP (CC = 0.472; *P*-value = 0.013); population density (CC = 0.747; *P*-value ≤ 0.001); number of inhabitants (CC = 0.396; *P*-value = 0.041); (iii) ICU beds at SUS and ratio of ICU beds to total beds (%) at SUS and the were correlated with the number of patients with COVID-19 (CC = 0.865; *P*-value ≤ 0.001); deaths due to COVID-19 (CC = 0.840); GDP (CC = 0.962; *P*-value ≤ 0.001); number of inhabitants (CC = 0.978; *P*-value ≤ 0.001) and % of the population (CC = 0.965; *P*-value ≤ 0.001).

The number of ICU beds at the private health system and the ratio of ICU beds to total beds (%) at the private health system were correlated with the number of patients with COVID-19 (CC = 0.911; *P*-value ≤ 0.001); deaths due to COVID-19 (CC = 0.909; *P*-value ≤ 0.001); GDP (CC = 0.930; *P*-value ≤ 0.001); population density (CC = 0.488; *P*-value = 0.010); number of inhabitants (CC = 0.901; *P*-value ≤ 0.001) and % of the population (CC = 0.896; *P*-value ≤ 0.001).

Finally, a positive correlation was observed between (i) the number of beneficiaries of private health insurance and the number of patients with COVID-19 (CC = 0.947; *P*-value ≤ 0.001); deaths due to COVID-19 (CC = 0.929; *P*-value ≤ 0.001); GDP (CC = 0.995; *P*-value ≤ 0.001); number of inhabitants (CC = 0.962; *P*-value ≤ 0.001) and % of the population (CC = 0.955; *P*-value ≤ 0.001).

In this context, the number of COVID-19 cases and epidemiological data in Brazil are correlated with the distribution of ICUs beds between regions. However, there is an important correlation between (1) the number of COVID-19 cases and deaths and (2) the need for private services and the number of beneficiaries of private health insurance (Table 4).

To corroborate with the previous findings, a partial correlation controlled by GDP and the number of beneficiaries of private health insurance was made, as follows: (i) ICU beds and the ratio of ICU beds to total beds (%) were correlated with population density (CC = 0.449; *P*-value = 0.024) and the number of inhabitants (CC = 0.414; *P*-value = 0.040); (ii) ICU beds/10,000 inhabitants was correlated with population density (CC = 0.681; *P*-value ≤ 0.001); (iii) ICU beds at SUS and the ratio of ICU beds to total beds (%) at SUS were correlated with the number of patients with COVID-19 (CC = –0.602; *P*-value = 0.001), deaths due to COVID-19 (CC = –0.609; *P*-value ≤ 0.001), number of inhabitants (CC = 0.688; *P*-value ≤ 0.001), % of the population (CC = 0.545; *P*-value = 0.005); (iv) ICU beds at SUS/10,000 inhabitants was correlated with the number of patients with COVID-19 (CC = –0.489; *P*-value = 0.013) and deaths due to COVID-19 (CC = –0.437; *P*-value = 0.029); (v) ICU beds at the private health system and the ratio of ICU beds to total beds (%) at the private health system were correlated with population density (CC = 0.604; *P*-value = 0.001) (Table 5).

Curiously, an inverse correlation occurred of ICU beds at SUS and the ratio of ICU beds to total beds (%) at SUS with the number of patients with COVID-19 and deaths due to COVID-19. Maybe, the data represents an association between the need to improve the public health system to treat COVID-19 in Brazil.

## Discussion

### Peculiarities to treat patients with COVID-19 in a vast geography territory: the example of Brazil

During the COVID-19 pandemic, the Brazil has taken steps to improve healthcare services mainly in the states with the highest number of COVID-19 cases, namely by constructing temporary field hospitals and facilitating access to the private health system. So far, a miracle drug to treat patients with COVID-19 or vaccine to control the pandemic have not been found [5,6,7]. The best choice of treatment for severe cases is intensive therapy and intubation. To perform the gold standard treatment, health services should have well-trained medical staff and infrastructure, including the availability of ICUs bed to all patients. Ultimately, Li et al. (2020) stated: “For a better understanding of this novel virus, more research needs to be done to get optimal strategies for the treatment of COVID-19” [7].

However, at Brazil, the availability of ICUs beds is unequal between states and Federal District. Brazil is a vast geographic territory with a population that is different in each region based on social behavior, genetics (each region has a different level of ancestral contribution of African, Caucasians and Indigenous genomes) and economic backgrounds raising the need for different medical and social managements in each area from Brazil. In addition, as showed in the descriptive and correlation analysis performed in our data, the number of patients with COVID-19 in need of public health system is higher than the private health care. Also, as stated by Ortega and Marson (2020),4 from the Influenza virus subtype (H1N1) pandemic (2009) to COVID-19 pandemic, Brazil lost ~34,500 hospital beds. In approximate numbers, hospital beds in the country fell from 460,920 to 426,380 in the short interval between the two pandemics, despite the increase in the number of inhabitants. The fall in the number of hospital beds occurred in the SUS, which lost 48,530 service spaces. In the same period, the private health service showed an increase of ~14,000 beds. However, Brazil gained 17,300 (from 42,400 to ~60,000–62,000 thousand) ICU beds in the same period.

Three Brazilian states [São Paulo (~18,000 ICUs), Rio de Janeiro (~7,000 ICUs) and Minas Gerais (~6,000 ICUs)] concentrated the ICUS and most of the ICUs are located in the capital cities [4]. The WHO recommends 1 to 3 ICUs beds for every 10,000 inhabitants and Brazil has approximately 1 bed for every 10,000 inhabitants, but the number of ICUs beds is unequal among different regions from Brazil including the adjustment by the number of inhabitants in each state. In addition, the number of equipment such as respirators is scarce, being, according to the government, ~65,000 respirators available in the country and, interestingly, in the H1N1 pandemic there were just over 35,000. The number presented here are unequal between states and the federal district. For example, Amazonas states has a vast territory comprising an area of 1,559,168 [12]. Km^2^ with a population of 4,144,597 inhabitants and the ICUs are concentrated only at Manaus (Capital city). To treat the severe cases for COVID-19 can be difficult in such situations because there is a limited access for mechanical ventilator, hospital, ICUs and, in many cases, transport to carry out the patients to the treatment site.

In Brazil, a special attention should be given for some groups of people as Indigenous people, health professionals and people living in urban low-income conglomerates namely as “Favelas” [4]. People living in favelas has precarious condition with, several times, no access to health, social and financial support. Also, people living at Favelas are susceptible to SARS-CoV-2 infection without the access to diagnose and/or treatment; and these people showed a low adhesion to the quarantine and social isolation. Obviously, considering this group of people, the biggest concern is the possibility of having the food to eat. Also, the houses are precarious one with many residents per unit in a small area.

Concomitantly, healthcare professionals need to be prepared for two other critical situations including the psychological effects of anxiety and depression due to death and other losses during the pandemic; [8] and the follow up and the treatment of diseases that were modified and postponed during the pandemic period, especially the respiratory diseases.

Moreover, Brazilian population comprise of ~500,000 indigenous citizens that has a limited access to hospital for intubation when needed [9]. Now, Brazil has several cases of indigenous with COVID-19 including 11,394 confirmed cases and 225 deaths [10]. The COVID-19 can be devastating for indigenous population mainly regarding the restrictions to have access to medical care. Also, the loss of indigenous ethnicities, in addition to characterizing the loss of life, reflects cultural and social loss that are irreparable.

Finally, as a vast geography territory Brazil show some peculiarities for some regions and can be affected by different pandemics simultaneously. For example, North of Brazil is at risk of Malaria and the outcomes for that is unknown [10]. There is no conclusion about if this zone (Amazon region) can be a hidden burden of COVID-19 due to Malaria symptoms or if is necessary to improve Malaria campaign and COVID-19 sensibilization to reduce the risk of both and concomitant infection. The Brazilian government and health authorities should implement health politics to control both diseases in an area with a vast geography dimension and several restrictions in the health system and with a population under poverty or extreme poverty condition with low access to the health system.

### Brazilian people and government actions to control the COVID-19 pandemic

There is a shortage of ICUs bed in many health services around the world. And, governments and health services are struggling to control the spread and infection rates. In this context, personal and environmental hygiene is key. However, the Brazilian population and even healthcare professionals are running low on medical supplies, such as facial masks and 70% alcohol [11]. Healthcare professionals, especially medical doctors, nurses and physiotherapists, are continuously working long hours and under intense pressure to treat all patients with COVID-19 in Brazil and worldwide [12,13,14]. Curiously, in Brazil, some health professionals are being harassed by some people who view the COVID-19 pandemic with disbelief or think that professionals can spread the disease.

In fact, some reactions of the population are associated with the position of President Jair Bolsonaro, who has minimized the risks of infection with SARS-CoV-2 and downplayed the severity of the pandemic in official nation-wide broadcasts, interviews and casual meetings with his supporters. In the midst of the coronavirus pandemic, Health Minister Luiz Henrique Mandetta’s stance towards the pandemic was in line with the WHO recommendations, but not in accord with President Bolsonaro’s position. Mr. Mandetta was eventually dismissed after Mr. Bolsonaro had publicly refused to abide by his social distancing guidelines. Also, in interviews and press conferences throughout February and March 2020, the Brazilian President reiterated the need for what he calls “vertical isolation” – isolation of the elderly and at-risk groups – to reduce the impact on economy. Furthermore, fake news around the disease and WHO recommendations spreads rapidly on social media: a new dilemma during the COVID-19 outbreak [15]. So far, social isolation and quarantine have been the best measures to reduce the number of SARS-CoV-2 infections, to offer the opportunity to treat all severe cases in ICUs and to reduce viral dissemination among less severe cases or asymptomatic patients [16,17,18,19,20,21].

Brazil shows a high number of healthcare professionals deaths affected by COVID-19 mainly infected at hospitals and ICUs. For example, as described by Palamim and Marson (2020) [22], on 20^th^ May 2020, the Federal Nursing Council from Brazil declared a total number of 138 (111 confirmed cases and 27 suspicious cases) deaths of nurses by COVID-19 representing 138/360 (38.33%) of all deaths for nurses worldwide. Also, the female sex represented 84.77% of the cases and 62.32% of the deaths. Moreover, the age range of infections was represented by: 20 to 30 years old – 2,938 cases (5 deaths); 31 to 40 years old – 6,849 cases (29 deaths); 41 to 50 years old – 24,456 cases (38 deaths); 51 to 60 years old – 1,485 cases (35 deaths); 61 to 70 years old – 201 cases (25 deaths); and 71 to 80 years old – 22 cases (6 deaths).

### The limitations to screen of SARS-CoV-2 RT-PCR on Brazil

During the onset of COVID-19 pandemic, a higher number of hospitalizations due to severe acute respiratory syndrome were registered in Brazil when compared with the number of previous years (2018 to 2019) [3]. However, the number of positive cases of COVID-19 announced by the government was not able to explain the higher rate of cases of severe acute respiratory syndrome. Therefore, the major problems are the limited access and capacity to screen the SARS-CoV-2 using RT-PCR. Although Brazil optimizes the test capacity day by day, approximately 296 tests per 1 million people had been performed. Until the confirmation of 2,000 deaths due to COVID-19, only 63,000 RT-PCR tests had been performed in Brazil. The low application of RT-PCR maybe increased the CFR because only severe COVID-19 cases are diagnosed. Keeping a low number of RT-PCR also increased disease dissemination because we failed to measure the number of less severe cases and asymptomatic individuals. This impasse could be solved with mass RT-PCR testing and environmental measures, such as tests to the detect SARS-CoV-2 tests in sewage [23,24,25,26].

As described in the literature, the factors that might be associated with the low number of SARS-CoV-2 RT-PCR tests carried out in Brazil include: (i) difficulty to purchase materials to perform the SARS-CoV-2 RT-PCR by the high market demand; (ii) increase in the price of materials and equipment to perform the SARS-CoV-2 RT-PCR; (iii) low availability of equipment; (iv) number of qualified people that are available to perform the RT-PCR technique; (v) number of centers or laboratories able to do the exam – in Brazil there are few test centers; and (vi) transportation of the material to the places where the test can be performed [27].

### Image exams to diagnosis patients with COVID-19

Lung high resolution computed tomography in patients with COVID-19 most commonly demonstrates ground-glass opacification with or without consolidative abnormalities, consistent with the diagnosis of viral pneumonia [28,29,30]. Lung high resolution computed tomography abnormalities are more likely to be bilateral, have a peripheral distribution, and involve the lower lobes [28,29,30]. Less common findings include pleural thickening, pleural effusion, and lymphadenopathy [28,29,30]. Lung high resolution computed tomography may be helpful in making the diagnosis. However, on Brazil and in some territories around the world, the lung high resolution computed tomography scan is not available to all individuals because lung high resolution computed tomography presents high cost, difficult accessibility for the patients affected, necessity of physical structure and patient transportation to the tomography equipment, exposure to radiation and lack of applicability during hospitalization.

As not all patients with COVID-19 can receive a lung high resolution computed tomography scan, especially in a rural zone, forest or Favelas. The lung ultrasound can be a feasible tool. Lung ultrasound is a tool used in lung pneumonia especially in low resource settings (especially tuberculosis) and in rare conditions as cystic fibrosis disease [31,32,33]. Thus, among the image exams, the lung ultrasound outstands and might become a useful tool to be used in the treatment and follow up of COVID-19 patients, mainly in more severe cases when intensive care is required. The lung ultrasound examination appears as an alternative in the respiratory system propaedeutic for being a low-cost technique, highly portable and that allows repetition of exams, and can be performed at the patient’s bedside [33].

The lung ultrasound in COVID-19 main characteristics are focal, multifocal and/or confluent B lines, corresponding to the ground glass opacity of the thorax lung high resolution computed tomography, in addition to the evidence of pleural thickening and irregularities. [34,35,36,37,38,39] Another advantage is the reduced need for manipulating the patient when compared to the lung high resolution computed tomography, avoiding the transportation of the patient to the X-ray room and reducing the risk of contamination of other patients and the health professionals directly or indirectly involved with the patient [34,35,36,37,38,39].

### Prevention to reduce the impact of COVID-19

Prevention is best practice in order to reduce the impact of COVID-19 pandemic considering the lack of effective treatment. At the moment, there is no vaccine available and the best prevention is to avoid exposure to the virus. In order to achieve this goal, the main measures, in Europe and Brazil, are the following: (i) to use face masks; (ii) to cover coughs and sneezes with tissues; (iii) to wash hands regularly with soap or disinfection with hand sanitizer containing at least 60% alcohol; (iv) to avoid contact with infected people; (v) to maintain an appropriate distance from people; and (vi) to refrain from touching eyes, nose, and mouth with unwashed hands.

Interestingly, the WHO issued detailed guidelines including (i) regularly and thoroughly clean hands with an alcohol-based hand rub or wash them with soap and water; (ii) avoid touching eyes, nose and mouth; (iii) practice respiratory hygiene covering your mouth and nose with your bent elbow or tissue when you cough or sneeze; (iv) if you have a fever, cough and difficulty breathing, seek medical care early; (v) Stay informed and follow the advice given by your healthcare provider; (vi) maintain at least 1 m (3 feet) distance between yourself and anyone who is coughing or sneezing.

In particular, regarding the use of face mask, health care workers are recommended by WHO to use particulate respirators such as those certified N95 or Filtering FacePiece 2 when performing aerosol-generating procedures and to use medical masks while providing any care to suspected or confirmed cases. Moreover, while an individual without respiratory symptoms is not required to wear a medical mask when in public, people with respiratory symptoms are advised to use medical masks both in health care and home care settings [40].

In Brazil, despite WHO recommendations, reports from the Ministry of Health and reports from state and municipal health departments, there is low adherence to the use of personal protective equipment and social isolation. In many places, crowds of people have occurred for several reasons, including: (i) meetings and parties of friends, colleagues and family; (ii) purchases in markets; (iii) visits to the bank, mainly by low-income people who will register or withdraw the emergency benefit provided by the Brazilian government in the amount of R$ 600 (~$100) to R$ 1,200 (~$200); (iv) demonstrations against and in favor of the federal government; (v) during visits by the president in numerous places in the national territory. In addition, the President of Brazil rarely uses personal protective equipment and during the interview to declare the positive diagnosis of the COVID-19, the Brazilian President removed his mask to prove to the population that he was healthy and would use chloroquine to control the manifestations of the disease.

## Management and therapeutic scheme for the SARS-CoV-2 infection on Brazil – Ministry of Health

### Protocol for the early supportive therapy and monitoring [41]

In Brazil, during therapy and support monitoring, oxygen therapy is indicated for patients with SARS and breathing difficulties, hypoxemia or shock. Conservative fluid treatment should be performed in patients with SARS when there is no evidence of shock.

Empirical antimicrobials to treat the probable pathogens that cause SARS can be administered and these antimicrobials must be provided within one hour of the initial assessment of patients with sepsis. Also, systemic corticosteroids to treat viral pneumonia or SARS outside clinical trials should not be administered routinely.

Patients with SARS for signs of clinical complications such as respiratory failure and rapidly progressing sepsis should be monitored, and supportive interventions should be applied immediately.

The patient’s comorbidities must be known so that individualized care and prognosis can be understood. Additionally, there must be good communication between the patient and his family.

### Protocol for the treatment of hypoxemic respiratory failure and acute respiratory distress syndrome – ARDS [41]

Severe respiratory distress should be recognized even when oxygen therapy is performed in high flow. In addition, mechanical ventilation should be instituted early in patients with respiratory failure with persistent hypoxemic (despite oxygen therapy).

In COVID-19, the use of non-invasive ventilation should be considered if respiratory discomfort is mild, if there is immunosuppression or if there is a diagnosis of cardiovascular problems. The non-invasive ventilation the should be done on negative pressure operating room. Additionally, endotracheal intubation should be performed if there is no response to noninvasive ventilation. The procedure must be performed by a trained and experienced professional, using aerosol precautions.

Mechanical ventilation should be implemented with the use of lower tidal volumes (6 (4 to 8) mL/kg of expected body weight) and lower inspiratory pressures (plateau pressure <30 cmH2O).

Patients with severe ARDS should be placed in a prone position to improve oxygenation, but patient safety must be guaranteed.

A conservative fluid management strategy should be adopted for patients with ARDS without tissue hypoperfusion. In addition, the patient must be disconnected from the ventilator, which results in loss of positive end-expiratory pressure and atelectasis. Inline catheters should be used for suctioning the airways and the endotracheal tube should be attached when it is necessary to disconnect the patient.

### Protocol for the management of septic shock [41]

Septic shock in adults should be recognized when infection is suspected or confirmed, and vasopressors should be used if necessary, to maintain mean arterial pressure above 65 mmHg and lactate above 2 mmol/L, in the absence hypovolemia.

Septic shock should be recognized in children with either hypotension (systolic blood pressure below the 5^th^ percentile or greater than 2 standard deviations below normal for age) or 2 to 3 of the following topics: altered mental status; tachycardia or bradycardia (heart rate <90 bpm or >160 bpm in babies and heart rate <70 bpm or >150 bpm in children); prolonged capillary recharge (>2 s) or hot vasodilation with bounding pulses; tachypnea; stained skin or petechial or purple rash; lactate increase; oliguria; hyperthermia or hypothermia.

In the resuscitation of septic shock in adults, administer at least 30 mL/kg of isotonic crystalloid to adults in the first 3 hours. In the resuscitation of septic shock in children in places with good resources, administer 20 mL/kg in rapid bolus and up to 40 to 60 mL/kg in the first hours.

Do not use hypotonic or starch-based solutions for resuscitation.

Administer vasopressors when shock persists during or after fluid resuscitation.

If central venous catheters are not available, vasopressors can be administered via a peripheral intravenous catheter, but a large vein must be used, and signs of local leakage and tissue necrosis must be monitored. If overflow occurs, the infusion should be stopped.

Vasopressors can be administered via intraosseous needles.

Consider the administration of intravenous hydrocortisone (up to 200 mg/day) or prednisolone (up to 75 mg/day) in patients with persistent shock who need increasing doses of vasopressors.

Dexethadone should be used in the dosage of 6 mg 1x day for 10 days in patients with the need for O2 support and the need for mechanical ventilation. Heparin 0.5 mg/kg 12/12h is also indicated for cases where there is no contraindication.

Drugs, such as chloroquine and hydroxychloroquine are provided can be supplied in Brazil, according to the Ministry of Health, in specific cases and through family consent.

### ICUs beds availability to treat patients with COVID-19 on Brazil

Our data give evidence that Brazil has a very diverse demographic and economic aspects. This diversity was also present in the states and the Federal District regarding the number of COVID-19 cases, deaths and CFR. The effective management of severe COVID-19 patients requires ICU services, and the scenario was also dissimilar as for ICU beds, ICU beds/10,000 inhabitants, ICU beds at SUS, ICU beds at SUS/10,000 inhabitants, ICU beds at the private health system, ICU beds at the private health system/10,000 inhabitants and beneficiaries of private health insurance during the onset of COVID-19 pandemic. The distribution of ICUs was uneven between public and private services, given that most patients rely on SUS, which has the lowest number of ICU beds. Moreover, in some states, the number of ICU beds at SUS was above 1 to 3 by 10,000 inhabitants, which was the number recommended by the WHO. RT-PCR testing is crucial to implement social policies to control the pandemic, but Brazil has failed to mass test the population. According to Marson (2020): “As human beings, we miss ‘that global hug’, which today is restrained and limited to our partners and in our homes. In a crisis situation, let us live light-heartedly and work together to make our world a better place” [42].
